# A controlled clinical study of periodontal health in anticoagulated patients: Assessment of bleeding on probing

**DOI:** 10.4317/jced.54331

**Published:** 2017-12-01

**Authors:** Pedro J. Almiñana-Pastor, Marta Segarra-Vidal, Andrés López-Roldán, Francisco M. Alpiste-Illueca

**Affiliations:** 1 DD, Post-graduated in Periodontics, Department d´Estomatologia, Facultad de Medicina y Odontologia, Universidad de Valencia, Valencia, Spain; 2 Department of Stomatology, School of Medicine and Dentistry, University of Valencia, Valencia, Spain; 3 MD DD, PhD in Medicine. Assistant Professor of Periodontics, Department d´Estomatologia, Facultad de Medicina y Odontologia, Universidad de Valencia, Valencia, Spain

## Abstract

**Background:**

According to the Spanish Society of Cardiology, 700,000 patients receive oral anticoagulants, and in these cases bleeding on probing (BOP) could be altered. However, no studies have analyzed the periodontal status of these patients and the effects anticoagulants may have upon BOP. A study was made of the possible relationship between plaque index, probing depth, INR (International Normalized Ratio) and acenocoumarol dose versus the clinical signs of BOP in a sample of anticoagulated patients. Likewise, an analysis was made of oral hygiene habits and attitude towards bleeding in these patients.

**Material and Methods:**

A controlled observational clinical study was made in La Ribera Hospital (Valencia, Spain) involving 44 anticoagulated patients treated with Sintrom® (acenocoumarol) and a homogeneous control group of 44 non-anticoagulated patients. A survey on oral hygiene habits and attitude towards bleeding was carried out, and the main periodontal parameters were recorded.

**Results:**

Probing depth was the parameter with the strongest correlation to BOP (*p*<0.001), followed by the plaque index (*p*<0.002). In contrast, no relationship was observed between acenocoumarol dose or INR and BOP. Mean BOP was greater in the control group than in the anticoagulated group (*p*<0.001). Oral hygiene habits and attitude towards bleeding differed significantly between groups.

**Conclusions:**

We have found no explanation why BOP was greater in the control group. What seems clear is that in the presence of the same plaque index and probing depth, anticoagulated patients did not bleed more than non-anticoagulated patients. A lack of knowledge of health and oral hygiene habits was observed in these subjects.

** Key words:**Anticoagulant therapy, bleeding on probing, periodontal health.

## Introduction

According to the Spanish Society of Cardiology, 700,000 patients receive oral anticoagulation therapy (OAT). The aim of such treatment is to prevent thromboembolic disease, one of the leading causes of disability and death beyond the fifth decade of life (1). The drugs used in OAT are coumarin derivatives known as vitamin K antagonists, since they are structural analogs of this vitamin. The most representative drugs in this category are warfarin (Aldocumar®) and acenocoumarol (Sintrom®). Both of them are derived from 4-hydroxycoumarin, and the differences between the two molecules are basically of a pharmacokinetic and pharmacodynamic nature (2).

From the pharmacodynamic perspective, both warfarin and acenocoumarol interfere with vitamin K metabolism, lowering the plasma levels of the vitamin K-dependent coagulation factors (Factors II, VII, IX and X) and of two natural coagulation inhibitors: proteins S and C. These drugs exert their effect within the liver cell microsomes, inhibiting the reductase required for transforming inactive vitamin K into active vitamin K (2).

The International Normalized Ratio (INR) is used to monitor anticoagulant activity. It expresses the ratio between prothrombin time (PT) and PT raised to the power of the International Sensitivity Index (ISI). In the case of a healthy individual not receiving OAT, the normal INR is between 0.9-1.1, while two therapeutic ranges are used in the case of patients subjected to OAT: the first INR range (2.5-3.5) applies to patients with mechanical valve prostheses, while the second range (2,3) applies to the rest of cardiac disorders (2).

The mean acenocoumarol dose is between 2-4 mg/day. With advancing age, dose reductions prove necessary. In addition to old age and polymedication, many other circumstances can account for the great variability in individual response to OAT, including genetic factors related to isoenzyme CYP2C9 (which mediates metabolization of the drug) and non-genetic factors such as a lack of adherence to therapy or dosing error; modifications induced by other concomitant drug treatments, herbal remedies or homeopathic products; changes in diet; or intercurrent medical diseases or other comorbidities such as diarrhea, among other alterations (2). Due to the great variability in treatment response, anticoagulated patients are subjected to periodic controls to determine the INR value and adjust the drug dose according to this parameter and other blood markers. In dental practice, many procedures can result in bleeding, including particularly tooth extraction and surgery. In such situations the bleeding risk would be increased in patients receiving OAT. The current recommendations are to not suspend anticoagulation in simple extractions and oral surgeries involving a low bleeding risk, provided the patient INR values are within the therapeutic range. In the case of more complicated surgery or in the presence of an increased bleeding risk, joint assessment of the case with the hematologist is required, and the suspension of OAT with the administration of low molecular weight heparin (LMWH) may prove necessary in some cases (3-6).

Importance of bleeding on probing (BOP)

Bleeding on probing (BOP) is a basic clinical exploratory sign used to evaluate gingival inflammation (7). Different indices are used for this purpose, though regardless of which of them is used, positive bleeding on probing is indicative of gingival inflammation. The diagnostic usefulness of BOP is undeniable, though its prognostic value is less clear. Many authors have concluded that the presence of BOP does not indicate an increased risk of destruction of tissue and supporting bone, i.e., this clinical sign has a low positive predictive value (8-10). From the perspective of periodontal treatment, however, it is essential to ensure healthy gums, without inflammation and therefore without BOP.

In 1991, Lang *et al.* (9) studied a group of 41 patients subjected to maintenance periodontal treatment, and found that in those zones where BOP proved positive, subsequent progression towards clinical attachment loss was seen in only 6% of the cases. In turn, in those zones where BOP proved negative, 98% of the cases remained stable. This study therefore underscored the high negative predictive value of BOP.

As has been mentioned, gingival inflammation is related to BOP. When a periodontal probe is inserted between the gums and the tooth (subgingival zone), the blunt tip of the probe comes into contact with the inflamed, thinned or ulcerated gingival epithelium, causing the rupture of small capillaries dilated as a result of the connective tissue inflammation (10). In view of the clinical importance of BOP, different factors that may influence this parameter have been analyzed with the purpose of standardizing this type of periodontal exploration. Factors inherent to the periodontal probe have been considered, such as the diameter of the tip of the instrument, its angulation (10,11) and the pressure applied on probing (12).

Smoking is another factor that can alter BOP, resulting in a decrease in the incidence of bleeding (13,14). In 2003, Nair *et al.* showed that smoking can induce false-negative results with the BOP test. In a group of 27 smokers these authors found the BOP rate to have doubled four weeks after smoking cessation (from 16% to 32%) (15).

Some investigators have described the effect of antiplatelet drugs upon BOP. In 2002, Schrodi *et al.* assessed the effect of acetyl-salicylic acid (Aspirin®) in patients without periodontal disease, and found a dose of 325 mg/day during one week to cause an increase in percentage BOP – particularly in individuals who previously presented BOP at 30% or more of the explored sites (16). Likewise, in 2004, Royzman *et al.*, in a sample of patients with gingivitis, found the administration of 81 to 325 mg/day of Aspirin® during one week to produce a significant increase in percentage BOP(14). It therefore can be concluded that antiplatelet medication is an aspect to be taken into account in the context of the patient anamnesis in order to correctly assess the true significance of BOP at periodontal exploration, since such medication may lead to error as a result of the associated increase in BOP. It may be inferred that anticoagulants, in the same way as antiplatelet drugs, act as BOP-modifying factors, though the pharmacodynamic characteristics of the two classes of drugs differ. However, despite the possible implication of anticoagulants as BOP-modifying factors, no data are available in the literature on the relationship between BOP and anticoagulant use. Likewise, no publications have been found on the oral health of patients subjected to OAT or their oral hygiene habits – these being very important factors particularly in patients of this kind, where prevention is a need rather than a preference, since the ultimate aim is to avoid more invasive procedures that might result in bleeding complications.

Oral anticoagulation therapy is the management of choice in certain cardiac disorders typically seen in the second half of life, and it is precisely in this age period when periodontal disease becomes more prevalent (17,18) and the conditions inherent to aging – such as diminished salivary flow and lowered host defenses – can lead to different kinds of oral diseases. All this and the lack of education in oral health among the more elderly population, as well as concerns about bleeding in these anticoagulated individuals, may contribute to worsen oral hygiene, as pointed out by Padrón *et al.* in 2003 (19). As a result, tooth extractions in patients subjected to OAT are quite frequent with the consequent healthcare costs and impact upon patient oral health. Furthermore, these subjects are at risk of suffering bleeding complications during or after the operation.

The aim of this study was to analyze the periodontal health, oral hygiene habits and attitude towards bleeding of a sample of anticoagulated patients, and to examine factors such as plaque index, probing depth, acenocoumarol dose and INR value, in order to determine whether OAT is implicated in BOP.

## Material and Methods

A controlled observational clinical study was carried out in a population sample of 44 individuals subjected to OAT with acenocoumarol (Sintrom®) and a homogeneous control group of 44 subjects without anticoagulation.

Patient screening. The study was carried out in the Department of Hematology of La Ribera University Hospital (Alzira, Spain). The patients were included on a consecutive basis in the order in which they were seen in the Department. The study was approved by the local Clinical Research Ethics Committee, and written informed consent was obtained from all the patients.

Inclusion and exclusion criteria. The study was limited to patients receiving acenocoumarol (Sintrom®), i.e., other forms of anti-coagulation such as subcutaneous low molecular weight heparin (LMWH) were excluded. Patients outside the 40-80 years age range were excluded, as were smokers or subjects who had stopped smoking less than a year ago, patients wearing removable dentures with fewer than 18 teeth in the mouth, individuals with more than two dental implants, and patients who had received periodontal treatment, systemic or local antimicrobials or any type of antiinflammatory medication in the three months before the exploration. Lastly, we also excluded patients receiving antiplatelet drugs or immunosuppressors, and pregnant or nursing women. These same exclusion criteria were applied to the control group, with the sole difference that these individuals were not receiving anticoagulants.

Study procedure and clinical recordings. The entire procedure and the recordings were carried out by the same individual blinded to the type of exploration. In a first phase we administered a questionnaire comprising basic questions on oral hygiene – some of a dichotomic (yes/no) nature and others involving short answers.

The daily dose of acenocoumarol (Sintrom®) administered to each patient was recorded, along with the INR value at the time of the survey. Following this first phase of the study, those patients that met all the inclusion criteria entered the second or periodontal evaluation phase. The plaque index was measured using a dental plaque revealer (Plac-Control®; floxin B 1.4%). We recorded the plaque scores for each tooth using the Quigley-Hein plaque index (1962) posteriorly modified by Turesky-Gilmore-Glickman (20), centering plaque measurement on the gingival third at three points of the buccal dental surface. After recording the plaque values in the periodontogram, all teeth present in the mouth were subjected to periodontal probing on the three buccal surfaces (mesiobuccal, medial and distobuccal).

**Figure 1:**
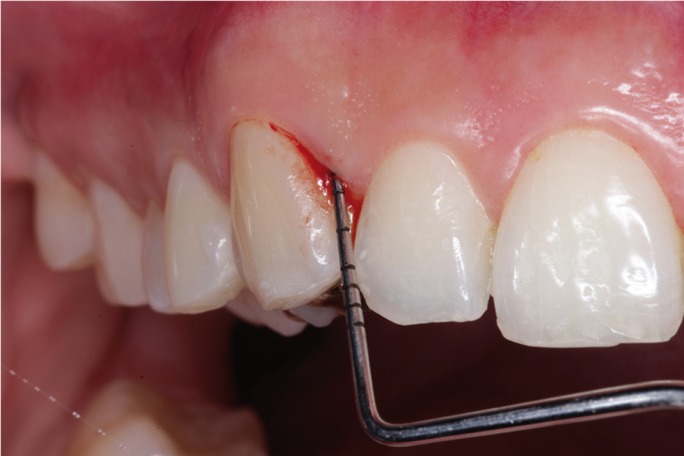
Bleeding on probing.

The following parameters were recorded in the periodontogram: probing depth, plaque index and the presence of BOP (Fig. 1). Bleeding on probing was considered positive when bleeding was observed 20 seconds after probing (21,22).

The same exploration was carried out in both the anticoagulated patients and the control group.

Statistical analysis

Descriptive, bivariate and multivariate analyses were performed in order to define a mathematical expression allowing us to account for BOP according to the rest of the periodontal parameters and the use or not of oral anticoagulant drugs. A multiple linear regression model was generated, with inclusion of all the factors in order to assess possible confounding effects and develop a fully adjusted final model. The usual applicability hypotheses were validated for the optimized model: normality of residuals, homoscedasticity, autocorrelation (Durbin-Watson) and non-colinearity. The accepted statistical significance level was 5% (α=0.05).

## Results

The final study sample consisted of 88 individuals divided into two groups: 44 anticoagulated patients and 44 controls. The mean age was 62.6 ± 9.7 years (range 40-80). The two groups were homogeneous in terms of age and plaque index ( Table 1).

**Table 1:**
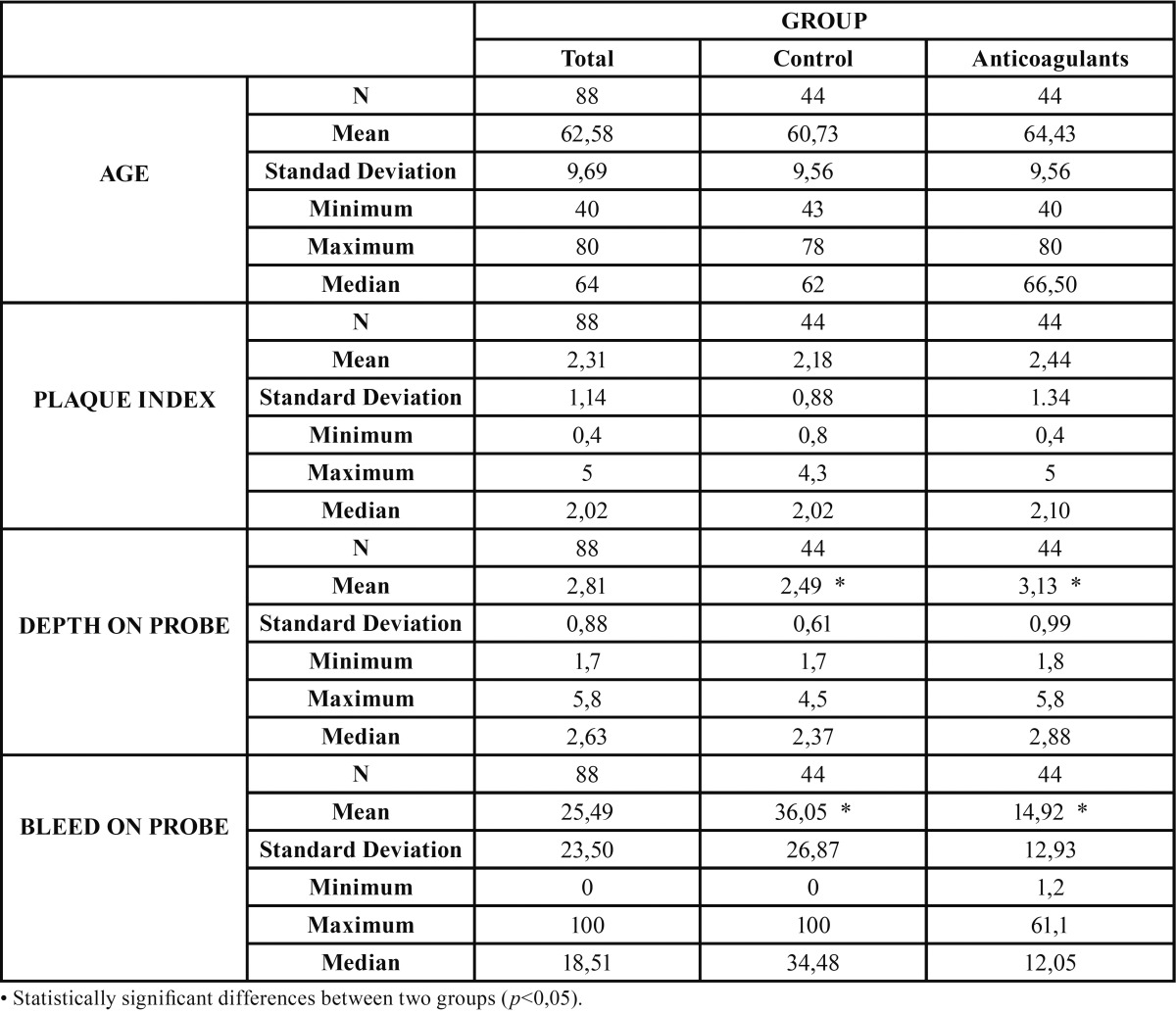
Homogeneity of control and test groups for age, plaque index, depth on probe and bleed on probe.

The mean probing depth differed significantly between the two groups, being greater among the anticoagulated patients than in the controls (*p*<0,001) ( Table 1).

-Bleeding on probing

**Figure 2:**
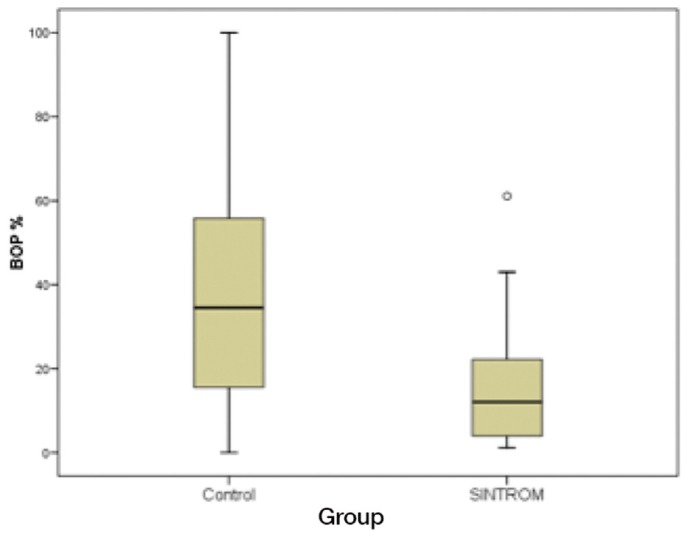
Box-plot showing BOP percentage in both groups.

The mean incidence of BOP was significantly greater in the control group than in the anticoagulated patients (36.05% versus 14.92%, respectively; *p*<0.001) (Fig. 2). Bleeding on probing was found to be significantly correlated (*p*<0.001) to both plaque index and probing depth. The multivariate linear regression model in turn showed probing depth to be the parameter with the greatest capacity to account for BOP (*p*<0.001). For each millimeter increase in probing depth, BOP was seen to increase 23.7%. This observation proved valid for individuals of the same group, plaque index, age and attitude towards bleeding. However, the level of significance between patient group and probing depth suggests that the relationship between BOP and probing depth differs according to whether anticoagulation is administered or not. The impact upon BOP was seen to increase with increasing probing depth in the control group. With regard to the plaque index, the findings were similar (*p*<0.002), and we moreover observed a strong group interaction tendency. For each additional point increment in the plaque index score, BOP was found to increase 9.9% on average. In effect, for a similar plaque index, the controls are expected to present a much higher BOP rate. All these conclusions related to bacterial plaque were also adjusted for the rest of the factors in the model, i.e., age and attitude towards bleeding.

-Questionnaire

As reflected by the results of the questionnaire, the oral hygiene conditions and attitude towards bleeding were seen to differ significantly between the two groups.

Interdental brushing was more frequent in the control group (27.3% versus 11.4% in the OAT group). Regarding the attitude towards bleeding, the anticoagulated patients were seen to be more concerned about bleeding: 65.9% claimed to fear bleeding in general (i.e., affecting any body location), versus only 9.1% of the controls. Furthermore, 29.5% of the anticoagulated patients claimed to be afraid of bleeding of the gums, versus only 13.6% of the controls. However, when asked about bleeding on brushing their teeth, 88.6% of the controls claimed to notice bleeding, versus 65.9% of the anticoagulated patients. On the other hand, in relation to fear of bleeding, 41% of the anticoagulated patients claimed to stop brushing when bleeding occurred, versus only 7% of the controls.

Another conclusion drawn from the questionnaire was that a larger percentage of controls were aware of the existence of perio-dontal disease and its treatment (18.4% versus only 5.4% of the anticoagulated patients).

Regarding the perceived cause of gingival bleeding, most of the anticoagulated patients either considered bleeding to be due to Sintrom® (34%) or were unable to specify the reason for bleeding (38.6%). In the control group, vigorous brushing (27.3%) and gingival inflammation (38.6%) were the main reasons given for gingival bleeding.

The results obtained are shown in  Table 2.

**Table 2:**
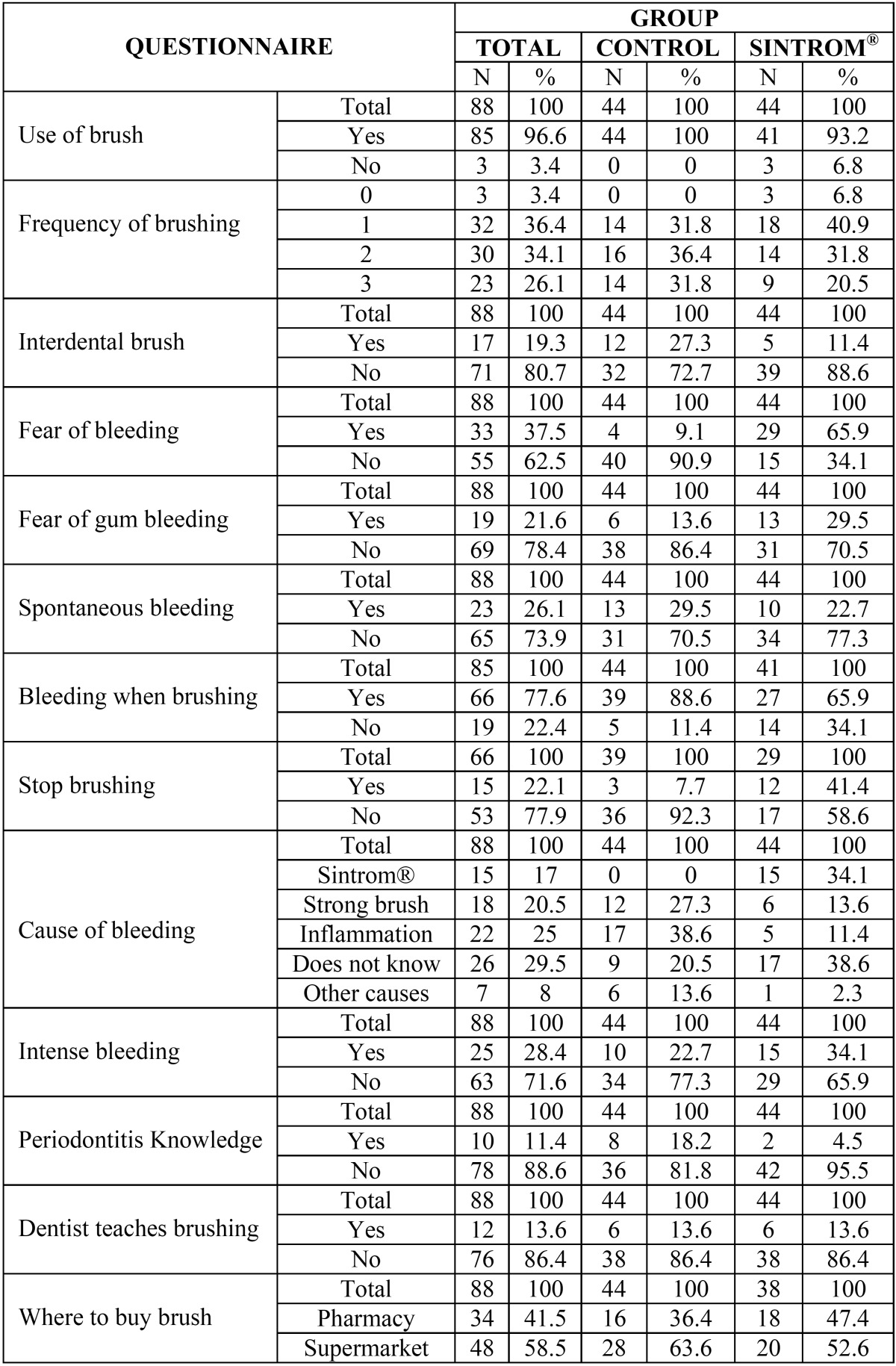
Analysis of the questionnaire on oral hygiene and fear of bleeding.

## Discussion

A curious finding of our study was that under homogeneous plaque index, probing depth and age conditions, the anticoagulated patients were seen to present less BOP than the controls. The explanation for this may reside in the possible effects of the anti-coagulant medication upon local inflammatory reaction; the possible concomitant diseases of the patients; interactions with other drug substances; or simply other factors not considered in our study.

In relation to the descriptive analysis of the results obtained in the OAT group, we attempted to correlate the Sintrom® dose and INR value to BOP. In the case of INR, no statistically significant association was found (*p*=0.809). Therefore, a greater or lesser INR value does not appear to be able to predict greater or lesser BOP during the clinical exploration of anticoagulated patients. On considering the drug dose, we found that for each additional milligram of acenocoumarol, percentage bleeding decreased 3.62%, i.e., the administered dose appears to be useful for predicting BOP (*p*=0.032). It cannot be accepted that an increase in acenocoumarol dose results in less BOP, due to the many factors that influence the dose administered to each patient and which have not been considered in our study, as well as to uncertainty regarding patient adherence to therapy. In this respect, a given patient may have a larger prescribed dose but fail to take the medication as indicated. In this case the dose may be further increased in an attempt to reach the optimum INR value, though BOP would remain clinically without change because of treatment non-compliance.

An association was found between the drug dose and INR value – the subjects with the highest INR also being the individuals with the highest Sintrom® dose, though statistical significance was not reached (*p*=0.57). It can be concluded that those patients with greater difficulty in achieving an optimum INR normally present higher and more irregular doses between successive controls.

We infer that bleeding would not be caused by acenocoumarol but by gingival inflammation. In pharmacodynamic terms, it could be postulated that BOP in principle should not be increased in anticoagulated patients, provided they are healthy from the periodontal perspective. The present study involving patients and controls matched for age, characteristics and dental plaque index was carried out to confirm this idea.

Schrodi *et al.* found that patients without periodontal disease administered 325 mg/day of Aspirin® during one week showed an increase in BOP, particularly among those individuals that already presented BOP of ≥ 30% of the surfaces (16). In turn, Royzman *et al.*, in patients with gingivitis, found that the administration of 81 to 325 mg of Aspirin® likewise produced an increase in BOP (14). Two key aspects should be considered on analyzing these two studies and comparing them with our own findings in anticoagulated patients. Firstly, the pharmacodynamic characteristics of acetylsalicylic acid (Aspirin®) are completely different from those of acenocoumarol (Sintrom®). Furthermore, as indicated by Schrodi *et al.* and Royzman *et al.*, BOP increases especially in those individuals that already present gingival inflammation (16,17). Adding the action of Aspirin®, which interferes with platelet plug formation, to this scenario would result in greater BOP. Probing depth is typically related to an increased plaque index, and both of these parameters are thus logically associated to increased BOP. In some cases, however, inactive periodontal pockets without BOP can be observed.

On comparing the anticoagulated patients versus the age- and plaque index-matched controls without anticoagulation, we found that although the former presented a greater mean probing depth and could thus be expected to present more gingival inflammation and hence greater BOP, these patients paradoxically exhibited less BOP than the controls. The descriptive analysis of the anticoagulated patients showed the Sintrom® dose to be negatively correlated to BOP. This result therefore may have been underestimated and wrongly attributed to chance. In any case, we have no plausible explanation for the abovementioned finding, though it may have been related to a decrease in local inflammation as a result of the action of the drug. A review of the literature has yielded no evidence supporting this idea, however. More objective data are therefore needed to clarify this association and discard a possible causal relationship dependent upon individual variability in response to OAT.

The main limitation of our study is the limited sample size. Another limitation refers to the fact that we did not measure the characteristics of BOP, i.e., we did not evaluate whether OAT was associated to more profuse or lasting bleeding as a consequence of interference with the coagulation process. Lastly, we did not use a periodontal probe of controlled pressure but only one experimented periodoncist explored all the patients.

With regard to the questionnaire ( Table 2), the anticoagulated patients expressed more fear of bleeding in general and of gingival bleeding. However, on comparing the data with those of the control group, we again made the surprising observation that 88.6% of the controls claimed to experience bleeding on brushing versus only 58% of the anticoagulated patients. It thus seems reasonable to postulate improved oral hygiene in the controls, despite their observed greater incidence of bleeding on brushing. Sixty-five percent of the anticoagulated patients did not consider the situation to be anomalous but rather a normal condition inherent to the oral cavity. These results suggest that the anticoagulated patients were less knowledgeable about the significance and relevance of gingival bleeding. The anticoagulated patients were seen to be unaware of the relationship between gingival inflammation and bleeding of the gums, and moreover did not know the cause of bleeding or attributed it to the use of Sintrom®. In contrast, the controls appeared to have greater awareness of the cause of bleeding.

The questionnaire was also used to investigate whether the anticoagulated patients were knowledgeable about periodontal disease and treatment. A surprising finding was the fact that 42 of the 44 subjects (95.4%) had never received periodontal treatment and were not familiarized with periodontal disease. On the other hand, only 6 of these patients (14%) had received instructions on oral hygiene from a dental professional.

The above observations point to the need for oral health education among anticoagulated patients. Oral health is to be promoted in patients subjected to OAT, since failure to do so may result in an increased number of tooth extractions and surgical procedures which logically imply bleeding risk and possible important alterations in INR secondary to either changes in anticoagulation allowing surgery (e.g., the administration of LMWH) or the postoperative high-dose analgesics and antiinflammatory drugs prescribed. Furthermore, surgery produces important patient stress, with changes in daily living habits after the operation. Strategies focused on tooth extraction should be avoided, placing emphasis on preventive measures and oral health promotion and education to prevent bleeding among anticoagulated patients undergoing surgery. Such measures not only lower the incidence of complications but also reduce the healthcare costs.

## Conclusions

1. The patients subjected to OAT with acenocoumarol (Sintrom®) had little knowledge about oral health, and this resulted in deficient periodontal conditions. The questionnaire revealed a lack of awareness of periodontal disease and treatment. Improvements are needed in this regard in order to prevent aggressive surgery and teeth extractions from becoming the management option of choice, since such treatments in patients receiving OAT result in important surgical stress and bleeding risk.

2. We have not found an explanation why BOP was higher in the control group. What seems clear is that with the same plaque index and probing depth, anticoagulated patients did not bleed more than non-anticoagulated patients.
